# Editorial: Molecular pathology in psychiatric diseases: frontiers of postmortem brain research

**DOI:** 10.3389/fpsyt.2023.1286182

**Published:** 2023-09-27

**Authors:** Yasuto Kunii, Mizuki Hino, Hiroaki Tomita

**Affiliations:** ^1^Department of Disaster Psychiatry, International Research Institute of Disaster Science, Tohoku University, Sendai, Japan; ^2^Department of Psychiatry, Tohoku University Hospital, Miyagi, Japan; ^3^Department of Psychiatry, Graduate School of Medicine, Tohoku University, Miyagi, Japan

**Keywords:** schizophrenia, bipolar disorder, neuropsychiatric disorder, genetic, molecular, postmortem brain

Biological studies, including genetic, physiological, imaging, and animal model studies, on psychiatric diseases, such as schizophrenia and bipolar disorder, have been actively conducted in various directions, and the elucidation of their pathophysiology is currently advancing rapidly. In addition, several studies at the molecular level using postmortem brains have recently been conducted to validate the findings of the above studies. Postmortem brain studies on schizophrenia, which were rarely reported until the 1970s, have increased drastically since the 1990s, and especially since the 2000s. This increase is a result of reevaluating the significance of brain pathology in schizophrenia, as the antipsychotic mechanism of action highlights the brain regions such as the midbrain, striatum, limbic system, and cortical tracts involved in the dopamine neurotransmitter system, and progress of structural brain imaging research using CT and MRI, and functional brain imaging research using SPECT and PET. Thus, postmortem brain research on schizophrenia and bipolar disorder is currently very active, and due to recent remarkable technological innovations in the field of basic neuroscience, examinations at the molecular level, such as analysis of mRNA (1), proteins (2–4) and lipids (5) expressed in the brain, and brain-specific genomic polymorphisms, such as CNV (6) and DNA methylation (7) are rapidly being conducted. Against the background of these multifaceted developments in neuroscience and the ability to perform comprehensive analyses of small amounts of tissue samples, postmortem brain studies on psychiatric disorders have shifted from the traditional role of validating the results obtained in basic neuroscience studies to a more exploratory role of adding new findings and providing feedback for other approaches. Therefore, if postmortem brain research is the final aggregation point for validating the findings accumulated in genetic, imaging, and animal model studies, it may indicate that our biological understanding of schizophrenia and bipolar disorder is entering its final phase.

In this Research Topic, we aimed to present cutting-edge postmortem brain studies on the molecular pathogenesis of schizophrenia and other psychiatric disorders and discuss the potential of these studies for clinical translation. This Research Topic highlights the recent advances in molecular pathogenesis, its relevance to clinicians, and potential for therapeutic development. Sano et al. focused on the molecular pathways implicated in lipid-related molecules, whereas Sano et al. and Hagihara et al. focused on dopamine-, glutamate-, and GABA-related molecules. Furthermore, Shishido et al. highlighted the recent findings on genetic risks, environmental stresses, and their interactions (G × E interactions). These reports also highlighted advances in bioinformatics based on comprehensive molecular profiling of the postmortem brain from patients with psychiatric diseases (e.g., cluster analysis and pathway analysis) (Sano et al.; Shishido et al.) and evaluation of the quality of postmortem brain resources from patients with psychiatric diseases (Miyahara et al.). Finally, the significance of integrating the data obtained from human postmortem brains with data from animal models of neuropsychiatric disorders was emphasized (Hagihara et al.). Up to the end of the call period, we collected four valuable original articles, which are presented in the next section.

Sano et al. conducted a trans-omics analysis of post-mortem brain samples from patients with schizophrenia and control subjects. Strong correlations were identified among the levels of phosphatidylinositol (PI) lipids, *TNC* mRNA, and APOA1 protein in the prefrontal cortex. PI (16:0/20:4) and *TNC* were positively correlated, whereas PI (16:0/20:4), APOA1, *TNC* and APOA1 were negatively correlated. PI (16:0/20:4) and *TNC* were decreased in the prefrontal cortex of patients with schizophrenia, whereas APOA1 protein was increased. Hagihara et al. used publicly available gene expression datasets to profile the expression patterns of pH-associated genes whose expression levels correlated with brain pH in human patients of 11 neuropsychiatric diseases such as schizophrenia, bipolar disorder, and Alzheimer's disease and mouse models of major central nervous system diseases, as well as in mouse cell-type datasets. They identified common and distinct patterns of gene expression in human disease and mouse cell-type datasets and found that some specific gene expression patterns were significantly correlated with the expression of genes related to GABAergic interneurons and glutamatergic neurons. Shishido et al. focused on stress as an environmental factor in stratified schizophrenia patients. They conducted a hierarchical cluster analysis of schizophrenia based on the gene expression levels of stress-responsive molecules in the postmortem prefrontal cortex, which yielded two clusters: a high-stress response group and a low-stress response group. They also performed a pathway analysis of differentially expressed genes (DEGs) between clusters and found that DEGs between clusters showed the highest enrichment for DNA double-strand break repair. Evaluating and controlling for confounders is necessary when investigating molecular pathogenesis in human postmortem brain tissue. Tissue pH and RNA integrity number (RIN) are valuable indicators when controlling for confounders. Miyahara et al. assessed its effects on gene expression in human brain tissue samples and analyzed the gene expression, while identifying gene sets affected by tissue pH and RIN. They also examined the functions of these genes by enrichment and upstream regulator analyses and found that genes commonly affected by tissue pH and RIN were highly associated with energy production and the immune system; genes uniquely affected by tissue pH were highly associated with the cell cycle, whereas those uniquely affected by RIN were highly associated with RNA processing. These findings will be helpful in controlling for confounders in future postmortem brain studies.

Taken together, these articles illustrate frontline postmortem brain research on the molecular pathogenesis of psychiatric disorders, particularly schizophrenia. One of the goals of postmortem brain research is to elucidate how specific neuronal circuits are altered in the disease state and develop new treatments that target these specific pathways. We anticipate that this Research Topic will be broadly informative for clinicians, physicians, and basic scientists interested in understanding the biology of psychiatric diseases.

## Author contributions

YK: Writing—original draft. MH: Writing—review and editing. HT: Writing—review and editing.
